# Comparison of the Effects of Left, Right, and Bilateral Carotid Baroreceptor Stimulation on Autonomic and Hemodynamic Responses Using an Indigenously Developed Paired Neck Chamber Device

**DOI:** 10.7759/cureus.82470

**Published:** 2025-04-17

**Authors:** Pratik Sorathiya, Kaushal A Desai, Prathamesh Kamble, Suchitra Dube, Anagha Sahasrabuddhe, Chaitali A Chindhalore, Mrunal Phatak, Ashwini Umredkar

**Affiliations:** 1 Mechanical Engineering, Indian Institute of Technology, Jodhpur, IND; 2 Physiology, All India Institute of Medical Sciences, Nagpur, IND; 3 Pharmacology, All India Institute of Medical Sciences, Nagpur, IND; 4 Radiodiagnosis, All India Institute of Medical Sciences, Nagpur, IND

**Keywords:** autonomic nervous system, baroreflex sensitivity, blood pressure regulation, carotid sinus stimulation, neck chamber device

## Abstract

Introduction

Baroreflex function plays a critical role in cardiovascular regulation, with carotid baroreceptors exerting a significant influence on autonomic and hemodynamic responses. While prior studies have suggested functional asymmetry between the left and right carotid baroreceptors, the findings remain inconsistent. These inconsistencies stem from several limitations, including small sample sizes, reliance on animal models, and inadequate consideration of confounding factors such as respiratory influence and baseline autonomic tone. A major barrier to conducting well-controlled human studies is the lack of non-invasive methods capable of delivering precise, side-specific baroreceptor stimulation. To address these gaps, we introduce a novel, non-invasive, digitally controlled paired neck chamber device that enables accurate, graded unilateral and bilateral stimulation using both negative and positive pressure, thereby overcoming several limitations of earlier techniques. The objective of this study is to evaluate the autonomic responses (RR interval (RRI) and heart rate) and hemodynamic responses (systolic blood pressure (SBP) and diastolic blood pressure (DBP)) to left, right, and bilateral carotid baroreceptor stimulation using graded negative and positive pressure stimuli. The study also seeks to evaluate baroreflex sensitivity (BRS) and the dose-response relationships under various stimulation conditions.

Methods

We conducted a prospective interventional study at All India Institute of Medical Sciences (AIIMS) Nagpur in collaboration with the Indian Institute of Technology (IIT) Jodhpur, involving 108 healthy young adults (57 males and 51 females). Participants had a neck ultrasound to find the carotid sinus, and then they were exposed to controlled pressure changes (from -100 mmHg to +100 mmHg) on the left, right, or both carotid sinuses. ECG and continuous blood pressure monitoring were used to evaluate autonomic and hemodynamic responses. Repeated measures using ANOVA analyzed variations in autonomic and hemodynamic responses among different stimulation sites. Nonlinear regression was employed to model dose-response relationships, while Friedman and Wilcoxon signed-rank tests were utilized to compare BRS gain. Multiple linear regression analyzed the relationships between BRS and markers of autonomic tone.

Results

Bilateral stimulation elicited the strongest bradycardic response (RRI up to 0.41 s) and more stable tachycardia under positive pressure compared to unilateral stimulation. Left-sided stimulation had a greater cardiac effect, while right-sided stimulation showed stronger blood pressure modulation (SBP and DBP fall up to 24.35 mmHg and 15.78 mmHg). BRS did not differ significantly across conditions, and the non-additive bilateral response suggests central integration.

Conclusions

This study provides new insights into carotid baroreceptor asymmetry, demonstrating that bilateral stimulation enhances autonomic modulation, while unilateral stimulation exhibits differential cardiac and blood pressure effects. These findings have clinical implications for baroreflex activation therapy (BAT) in conditions such as hypertension and heart failure. Future research can explore long-term adaptations, neuroplasticity, and central baroreflex processing to refine therapeutic strategies.

## Introduction

The autonomic nervous system plays a critical role in maintaining cardiovascular homeostasis through baroreceptor reflexes, which regulate heart rate and blood pressure in response to changes in arterial pressure. The carotid baroreceptors, located in the carotid sinus, are essential components of this reflex mechanism. When stretched, these mechanoreceptors are stimulated and transmit afferent signals to the brainstem, modulating sympathetic and parasympathetic activity to maintain hemodynamic stability [1,2]. Baroreflex sensitivity (BRS) is a key physiological measure used to assess autonomic function, particularly in the context of cardiovascular health. Altered BRS has been implicated in various clinical conditions, including hypertension, heart failure, and autonomic dysfunction [3]. Traditional methods for assessing BRS include pharmacological interventions, spontaneous sequence analysis, and mechanical neck chamber techniques that induce changes in carotid sinus pressure (CSP) [4]. Conventional approaches for evaluating BRS encompass pharmaceutical interventions, spontaneous sequence analysis, and mechanical neck chamber methodologies. Most of these approaches can't stimulate the baroreceptor unilaterally. Consequently, they are unable to distinguish between left, right, and bilateral carotid baroreceptor sensitivities. As a result, research on the functional asymmetry and integrative mechanisms of the baroreflex system is limited. These insights are significant, as the findings have implications for emerging medicines, such as baroreceptor-based neuromodulation therapies, including baroreceptor activation therapy (BAT), for diseases like hypertension and heart failure.

The left carotid baroreceptor is generally believed to exert a more significant effect on vagal modulation of heart rate, while the right carotid baroreceptor is thought to primarily govern sympathetic vasomotor tone [5]. These differences may be due to anatomical and functional asymmetries, including receptor density, vascular wall compliance, and lateralized central integration pattern in the nucleus tractus solitarius (NTS) [5]. The magnitude of these differences is ambiguous, and there is a paucity of comparative evidence about the effects of left, right, and bilateral stimulation on autonomic and hemodynamic responses; additionally, the findings are inconsistent. These inconsistencies largely stem from methodological limitations, especially the lack of widely available non-invasive techniques for precise, side-specific baroreceptor stimulation in humans. Much of the existing evidence is derived from animal studies, which indicate that the right carotid baroreceptors have a more significant impact on heart rate modulation than the left [6,7]. Kawada et al. showed no significant change in cardiac efferent sympathetic activity between left and right carotid stimulation in a rabbit model [8]. There are important limitations in translating animal research findings to humans, including species-specific differences in baroreceptor anatomy, baseline heart rate ranges, and central autonomic processing. Additionally, animal studies are often conducted under controlled laboratory conditions that do not reflect the variability in human physiology with respect to resting autonomic tone and individual response patterns. These differences underscore the need for well-controlled human studies using techniques that can isolate and compare left, right, and bilateral carotid baroreceptor stimulation. Investigations into carotid baroreceptor stimulation in humans have produced inconclusive results. Some research suggests right-sided dominance in cardiac regulation and blood pressure management [9,10]; however, another study revealed that left carotid sinus stimulation provoked more pronounced muscle sympathetic nerve activity (SNA) responses [11]. Conversely, some studies have demonstrated an absence of notable asymmetry in baroreflex function [12]. Electrical stimulation studies, especially in hypertension patients, support unilateral right-sided BAT for optimal results [13]. These results stem from prior research with limitations. Human studies exhibit limited sample sizes (ranging from nine to 30 individuals) and demonstrate an uneven gender distribution. The impact of breathing on baroreflex modulation has been overlooked, and baseline autonomic tone has not been considered or adjusted in most of the studies. The technique for carotid stimulation also differs in all the studies. The studies involving neck collar devices primarily employ sinusoidal waves at defined frequencies, concentrating on negative stimulation, while positive stimulation for the neck chamber remains largely unexamined. Addressing these gaps requires more comprehensive and standardized research approaches.

The present study utilizes an indigenously developed paired neck chamber device that can administer regulated graded pressure stimulation to the left, right, or both carotid baroreceptors concurrently. This newly developed approach is based on the mechanical neck collar technique [14]. This technique used for evaluating BRS entails administering regulated pressure alterations to the carotid sinus area, mimicking normal variations in arterial pressure. This non-invasive technique enables real-time assessment of baroreceptor function without the use of pharmacological agents. The existing neck collar techniques for BRS assessment, which range from bulky chamber boxes to simplified paired earphone-based designs [14-16], face practical limitations such as discomfort, incomplete stimulation, and the need for repeated tests. These issues underscore the necessity for a more ergonomic and efficient alternative. In our prior publication [17], we constructed a neck chamber device capable of unilateral stimulation of the carotid baroreceptors. The preliminary prototype included a single chamber for unilateral stimulation, functioning entirely through manually operated pumps. Pilot testing with this prototype demonstrated a baroreceptor gain of -0.109±0.04, which is consistent with values reported in earlier studies using neck collar devices, thereby supporting its physiological validity [17]. In the present study, we have upgraded the device by incorporating paired neck chambers for bilateral carotid baroreceptor stimulation, integrating digital operation, and developing a desktop-based interactive computer program to regulate the stimulation process.

We hypothesized that bilateral carotid baroreceptor stimulation would elicit stronger and more stable autonomic and hemodynamic responses compared to unilateral stimulation, and that left- and right-sided stimulations would produce distinct effects on heart rate and blood pressure, respectively, due to functional lateralization of baroreceptor pathways.

The primary objective of the present study was to assess and compare autonomic reactions (RR interval (RRI) and heart rate) and hemodynamic responses (systolic blood pressure (SBP) and diastolic blood pressure (DBP)) during unilateral (left and right) and bilateral carotid baroreceptor stimulation by graded negative and positive pressure stimuli using an upgraded neck chamber device in healthy subjects. The secondary objectives were to evaluate and compare dose-response relationships and maximum BRS across left, right, and bilateral carotid sinus stimulation. In this study, the term “dose-response” refers to the graded physiological effects (heart rate and blood pressure changes) elicited by increasing intensities of mechanical pressure stimuli delivered to the carotid sinus, rather than pharmacologic dosing.

## Materials and methods

This prospective interventional study was conducted at the Department of Physiology, All India Institute of Medical Sciences (AIIMS) Nagpur, in collaboration with the Department of Mechanical Engineering, Indian Institute of Technology (IIT) Jodhpur.

Design and development of the upgraded paired neck chamber

**Figure 1:**
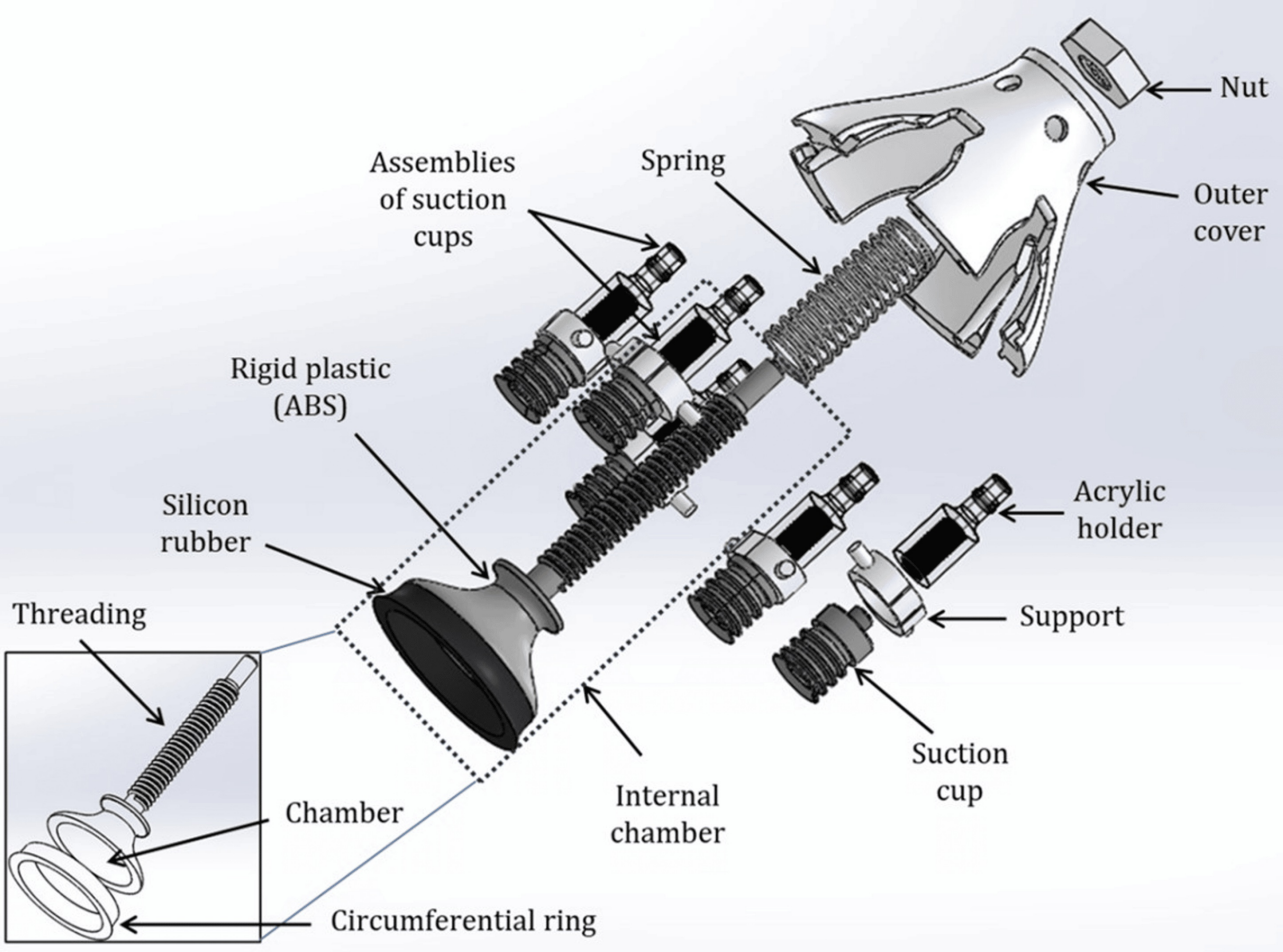
Exploded view of the mechanical assembly of the paired neck chamber device for non-invasive carotid baroreceptor stimulation This figure illustrates the detailed mechanical assembly of the paired neck chamber device used for controlled, non-invasive stimulation of the carotid baroreceptors. Image Credits: Authors

This study provides an upgraded version of the previously published neck chamber device [17], in terms of incorporating paired neck chambers for bilateral carotid baroreceptor stimulation, integrating digital operation, and developing a desktop-based interactive digital utility to regulate the stimulation process. The newly designed device applies controlled pressure within a predefined range to stimulate the carotid baroreceptors using a square wave method. The pressure varies from −100 mmHg to +100 mmHg in increments of 20 mmHg. The device comprises a pressure source, neck chamber, and pneumatic logic controller. The pressure source includes a low-pressure generator unit to regulate the controlled pressure in the chamber. The neck chamber device includes an internal chamber and an outer suction assembly. The outer cover is attached to the internal chamber and connected with a suction assembly. The internal chamber includes threading a circumferential ring within the chamber. The circumferential ring is connected to the chamber with the outer threading. The unit generates both positive and negative pressure cycles on the skin. The unit was fabricated using 3D printing with a circumferential ring with a low Shore hardness value (Shore 40) to ensure attachment to the skin surface making it easily indentable and capable of conforming to the skin surface for secure attachment and minimal air leakage. The outer suction assembly ensures securing the entire neck chamber device to the skin (see Figure 1).

**Figure 2:**
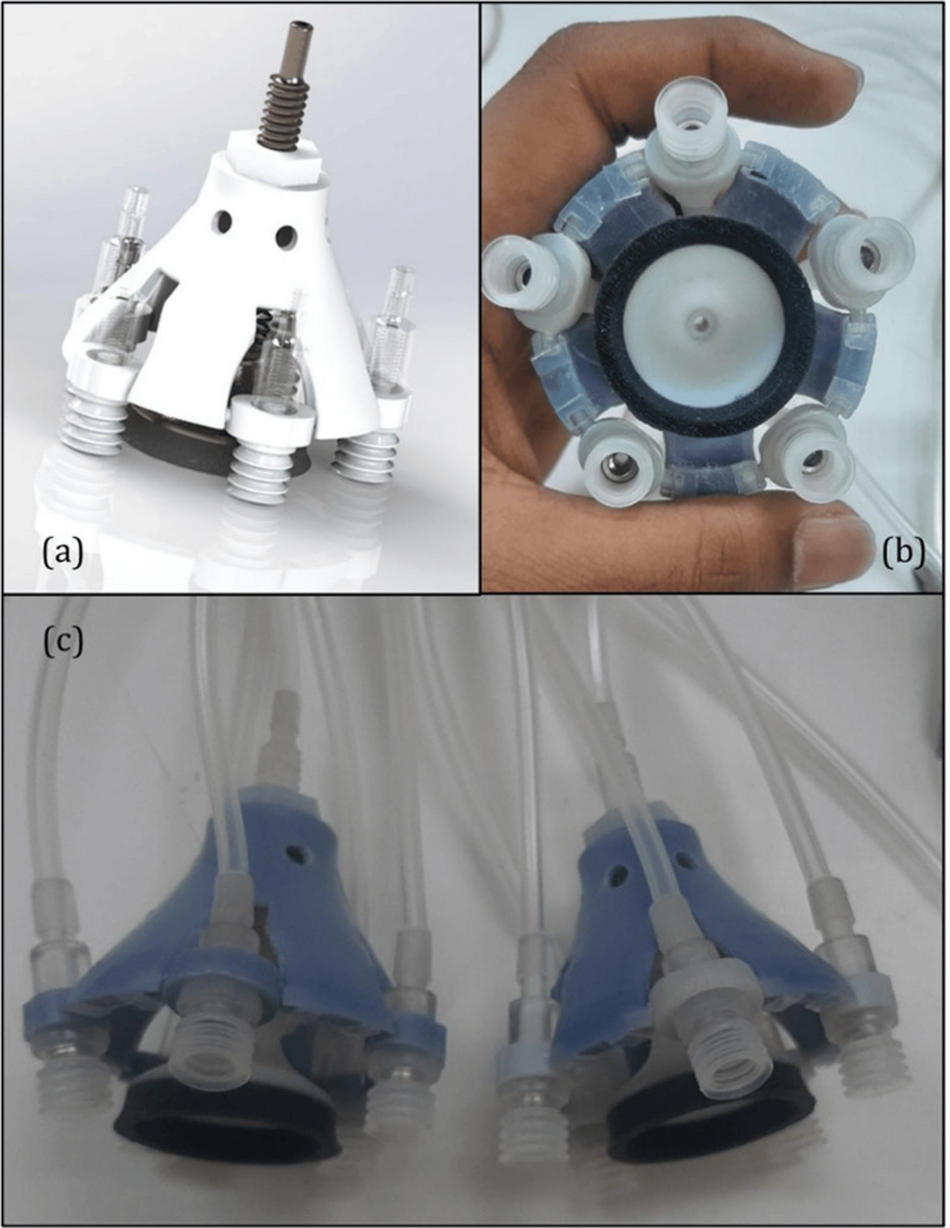
Structural configuration of the paired neck chamber device for non-invasive carotid baroreceptor stimulation (a) CAD model of the neck chamber device, showing its outer structure, suction components, and spring mechanism. (b) Top-down view of the assembled device, highlighting the inner and outer chambers and the suction cup, which ensures optimal skin contact and pressure transmission. (c) Paired neck chamber device setup, demonstrating its configuration for bilateral carotid baroreceptor stimulation. The tubing and connectors facilitate controlled pressure modulation for experimental and clinical applications. CAD, computer-aided design Image Credits: Authors

The outer assembly consists of three components: an acrylic holder, a support structure, and a suction cup. The suction cup is made of silicone rubber and is located at the bottom of the assembly to ensure a firm grip on the skin. The support structure is made using 3D printing and secured with an acrylic holder. The outer suction assembly is housed within the outer cover, as shown in Figure 1. The outer suction action is performed initially to ensure that the neck chamber device is adhering to the subject. The internal chamber unit generates predefined pressure over the skin using electro-pneumatic logic control. The neck chamber device is connected to the electro-pneumatic logic unit via tubing, as shown in Figure 2.

**Figure 3:**
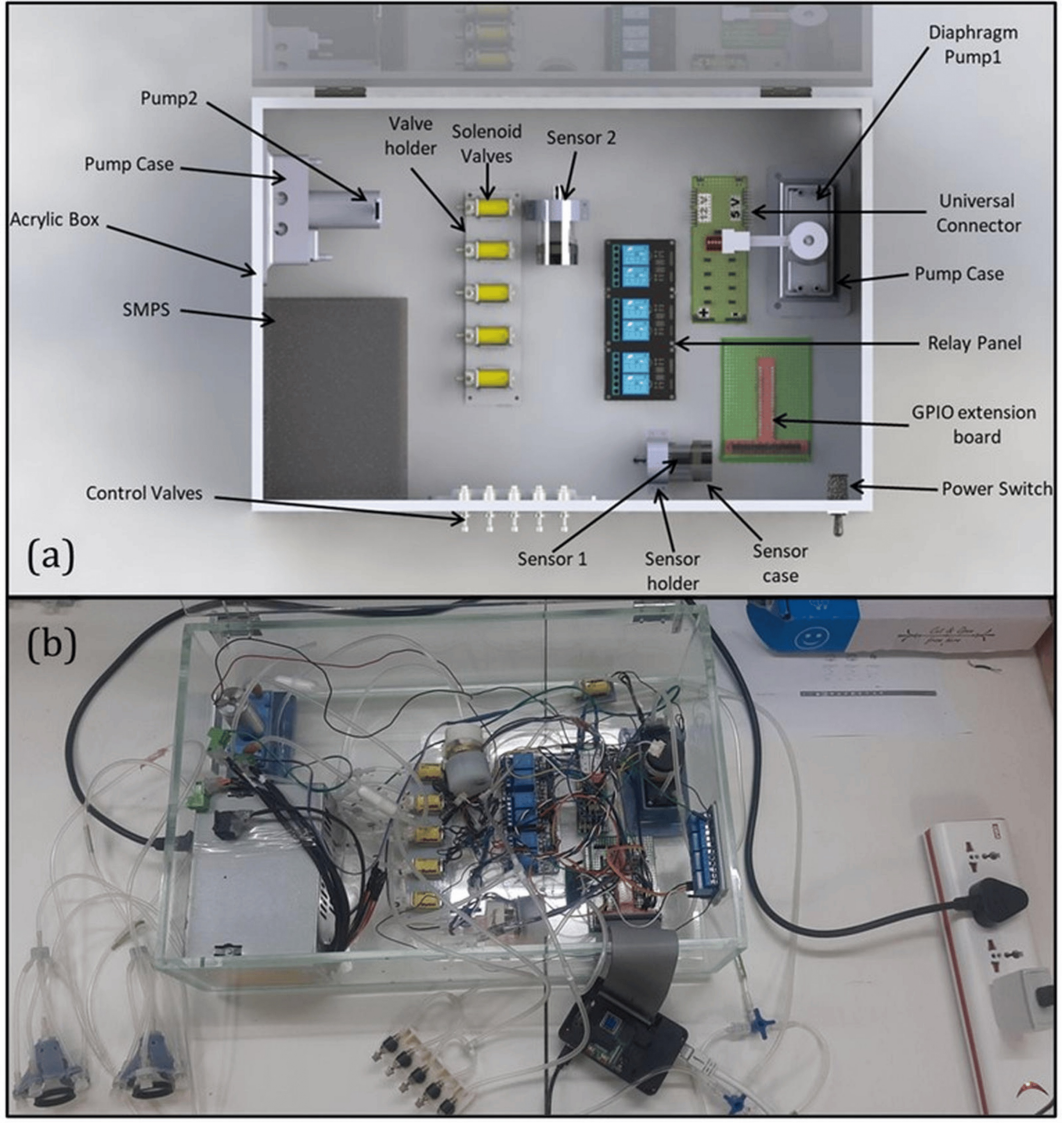
The electro-pneumatic logic control unit used for automated pressure modulation in the paired neck chamber device for carotid baroreceptor stimulation (a) CAD model of the electro-pneumatic logic control unit, highlighting key components such as solenoid valves, diaphragm pumps, control valves, relay panel, and GPIO extension board. These components regulate the pressure applied to the paired neck chambers. (b) System arrangement of the assembled unit, showing the real-world implementation of the device with wired connections, power supply, and control mechanisms integrated for experimental use. Image Credits: Authors CAD, computer-aided design

The system allows two option modes: a single or a paired chamber device. The chamber is linked to the pneumatic logic control to provide adjustable pressure that activates carotid baroreceptors and sticks to the skin using a vacuum grip system. The pneumatic logic control unit manages the pressure inside the chamber and the external gripping unit. The electro-pneumatic logic system is controlled using an indigenously designed graphical user interface (GUI). Figure 3 shows the electro-pneumatic logic unit that can perform positive and negative pressure cycles with sensor measurements.

**Figure 4:**
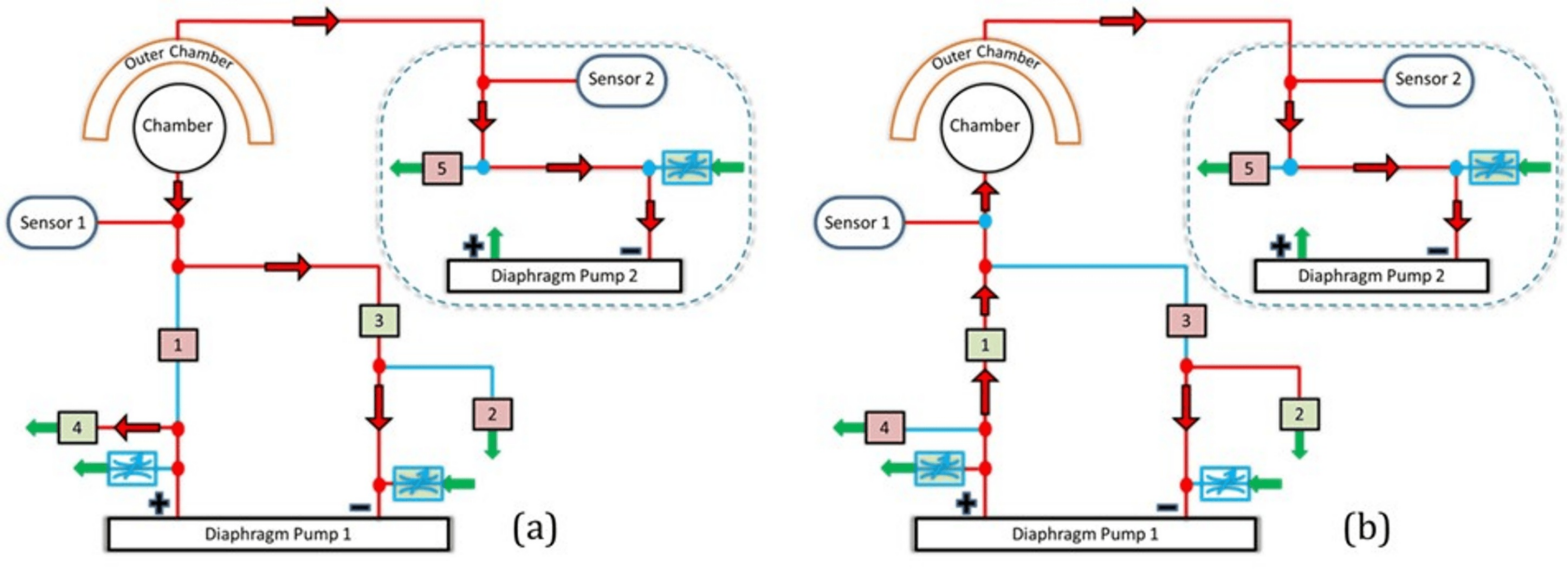
Electro-pneumatic logic circuit for pressure regulation in the paired neck chamber device (a) Negative pressure cycle: Air is drawn out of the chamber by Diaphragm Pump 1, creating a suction effect that activates the carotid baroreceptors. The control system, including valves (1, 3, 4), sensors (1, 2), and Diaphragm Pump 2, ensures precise pressure modulation. (b) positive pressure cycle: Air is introduced into the chamber, applying controlled compression on the carotid sinus. The pressure delivery is regulated by the same system components, with reversed airflow direction through valves (1, 2, 3, 5) and Diaphragm Pump 2 assisting in maintaining stability. Image Credits: Authors

The system includes a solenoid valve panel responsible for the pneumatic logic. A pressure source connects the valves to a miniature diaphragm pump. The system uses two diaphragm pumps arranged in series to form a dual-head configuration, each serving a distinct purpose. The first pump is responsible for delivering positive and negative pressure (up to ±400 mmHg) to the inner chamber, which directly stimulates the carotid baroreceptors. The second pump generates negative pressure in the outer chamber, which serves as the gripping mechanism, securing the device firmly against the skin during stimulation. This design allows different pressure levels to be used at the same time in the stimulation and gripping chambers, ensuring accurate activation of the baroreceptors while keeping a steady contact and reducing any leaks or loss of pressure. The pump operation (ON/OFF) is controlled using a Raspberry Pi controller and a relay switch. This system was chosen for its cost-effectiveness, flexibility, compact design, and ease of programming and integration with various sensors and GUI-based control modules. This setup supports customizable, real-time control over the timing and magnitude of pressure delivery, offering a scalable platform for future iterations of the device. Two sensor modules are integrated into the pneumatic logic circuit for pressure regulation. One sensor monitors the pressure in the chamber, while the other monitors the pressure of a gripping mechanism. The sensors had an accuracy of ±1 mmHg, which is well within the physiological pressure modulation range used in the study (−100 mmHg to +100 mmHg). This level of precision ensured reliable monitoring and feedback control, supporting the validity of the stimulation protocol and the reproducibility of the pressure cycles. The electro-pneumatic logic circuits for the positive and negative pressure cycles are shown in Figure 4. 

**Figure 5:**
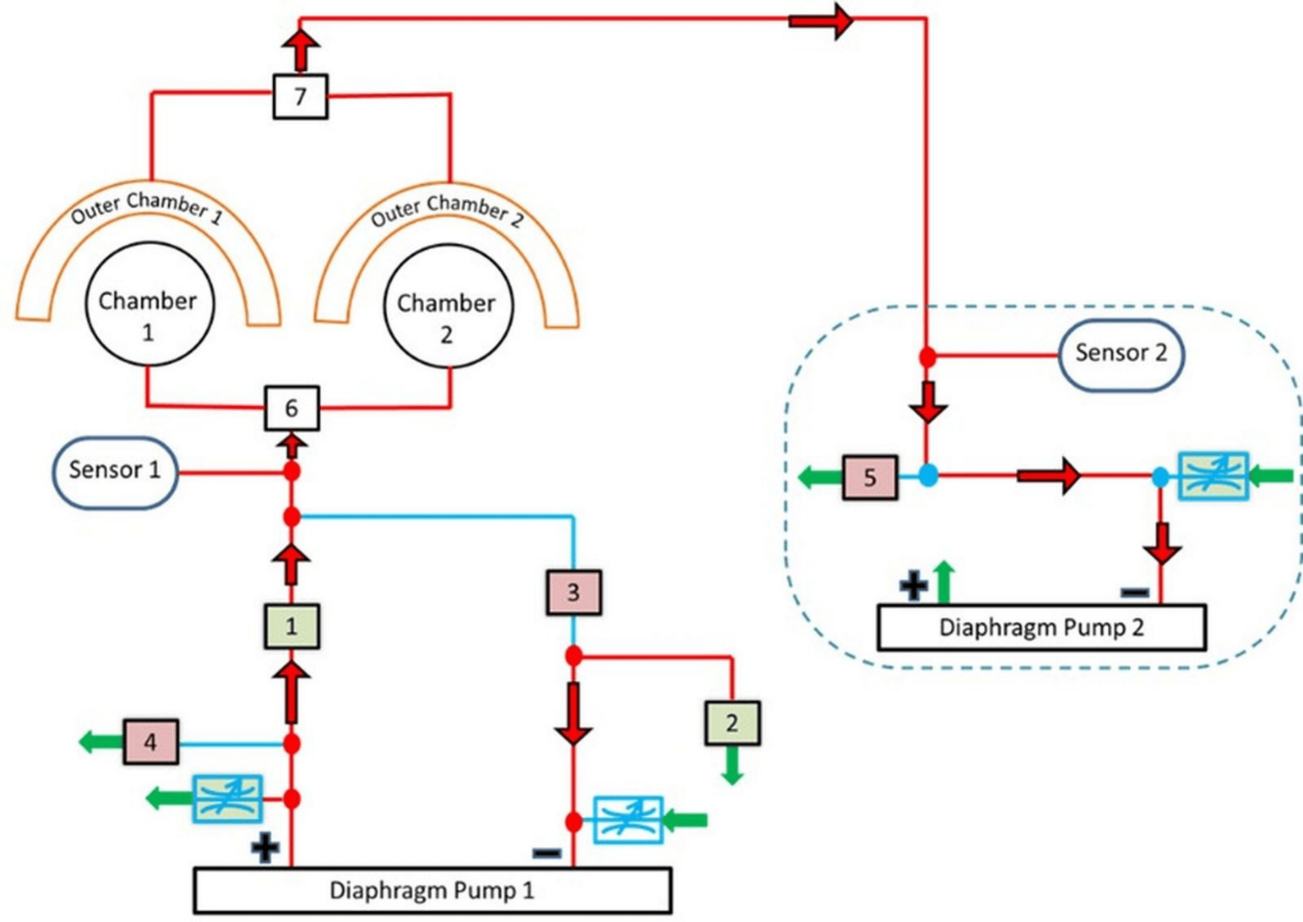
The electro-pneumatic logic circuit used to regulate simultaneous bilateral pressure stimulation in the paired neck chamber device for carotid baroreceptor activation Image Credits: Authors

The red arrows in the diagram indicate airflow directions to the chamber and suction cups. The square boxes represent solenoid valves, with the red box indicating a closed valve and the green box representing an open valve. The airflow direction can be reversed using a solenoid valve. The blue boxes represent manually adjusted flow control valves to achieve the desired pressure within the internal chamber. During the negative cycle, air flows from the chamber to the inlet of Diaphragm Pump 1 and is exhausted to the atmosphere through Solenoid Valve 4. In the positive pressure cycle, pressurized air flows from the pump outlet to the internal chamber when Solenoid Valve 1 is open and Valve 4 is closed. The pressure transition time between set points was typically within 1 second, allowing for rapid and consistent modulation of baroreceptor stimuli. The electronically controlled valves and programmable microcontroller allowed for accurate timing and quick responses, with no noticeable effect on the physiological data from any delays. Figure 5 shows the operation of a paired configuration set using the T-valves.

**Figure 6:**
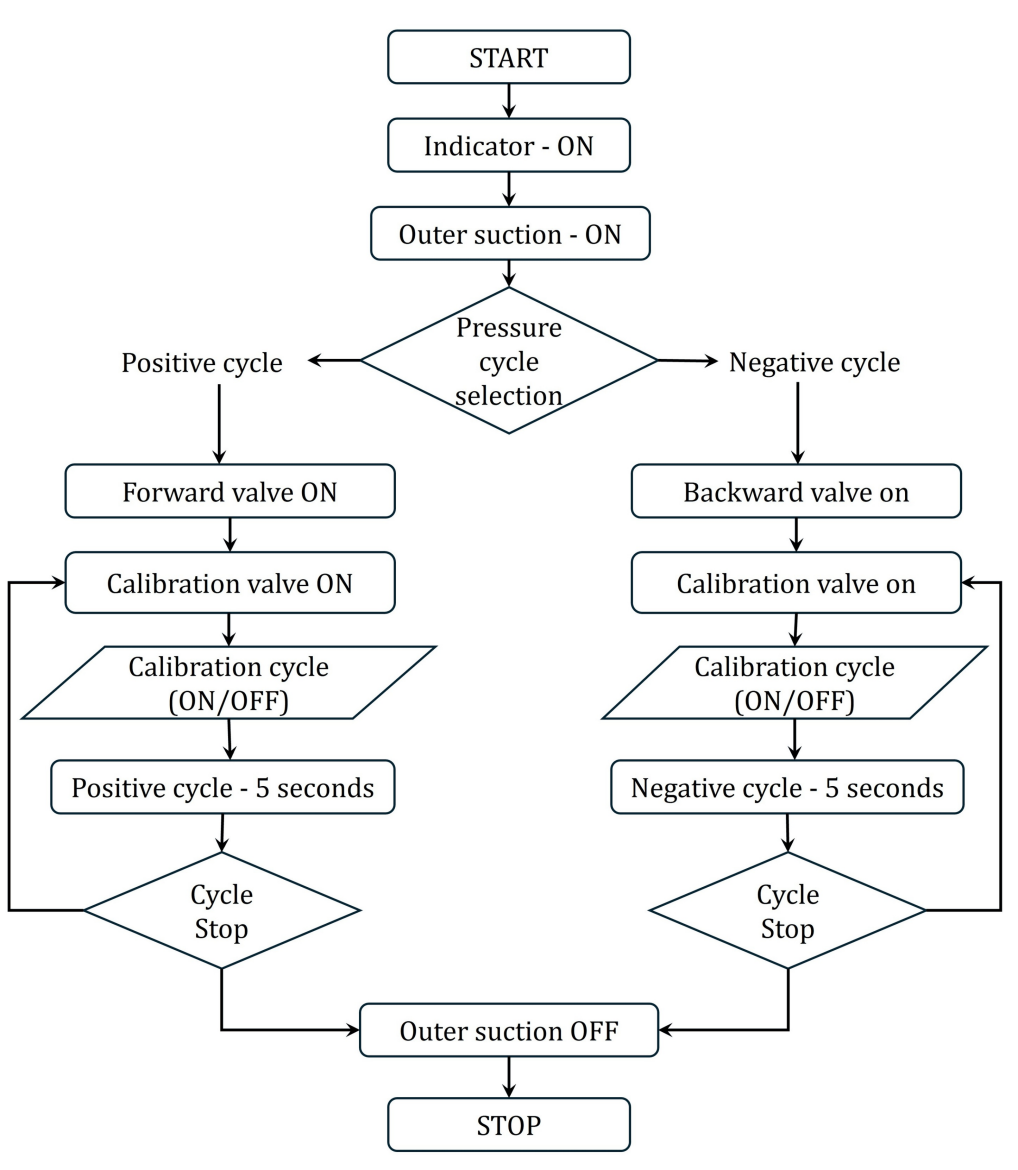
The operational sequence of the neck chamber device used for controlled, non-invasive carotid baroreceptor stimulation Image Credits: Authors

Figure 6 illustrates the flow diagram, providing a clear overview of the sequence of operations.

The system operation commences with the positioning of the chamber device on the surface of the human skin. After attaching the neck chamber device, the outer suction cycle activates to generate negative pressure, which facilitates the attachment of the device to the skin. The electro-pneumatic logic unit gradually applies the internal chamber pressure, either positive or negative, to ensure controlled stimulation. For bilateral stimulation, both neck chamber devices are turned on at the same time using direction control valves to work together.

Comparison of BRS for laterality of response

The testing of the upgraded paired neck chamber device was carried out in the Department of Physiology at AIIMS Nagpur after approval from the Institutional Human Ethics Committee. Informed written consent was obtained from all participants prior to their involvement in the study, in accordance with the Declaration of Helsinki.

The study was conducted on 108 healthy young volunteers, with a mean age of 22.92 ± 1.33 years, comprising 57 males and 51 females. The participants had an average height of 164.9 ± 11.33 cm, weight of 65.58 ± 5.25 kg, and BMI of 23.80 ± 2.8. Before the study, all details were thoroughly explained to the participants, and written informed consent was obtained. Volunteers were selected based on predefined inclusion and exclusion criteria. Healthy males aged 18 to 30 years with no history of cardiovascular, respiratory, neurological, or metabolic disorders were included. An upper BMI limit of <28 kg/m² was enforced during recruitment to exclude individuals with higher cardiometabolic risks. All participants were nonsmokers and non-alcoholics. Additionally, none were on any medication that could influence autonomic function or cardiovascular physiology. Exclusion criteria included a history of hypertension, diabetes, or chronic illness. Individuals who had experienced illness within the past month, engaged in intense physical training, or participated in professional athletic activities were also excluded. Rigorous training is known to alter baseline autonomic tone and BRS, which could skew results and reduce generalizability to the healthy population. Volunteers with skin lesions, infections, or anatomical abnormalities at the carotid sinus region that could interfere with device placement were excluded. Furthermore, individuals with a history of syncopal episodes or autonomic dysfunction were excluded from participation.

Participants were instructed to avoid eating for at least two hours and abstain from caffeine (coffee or tea) for 24 hours prior to testing to reduce variability in autonomic tone and cardiovascular responses that could confound BRS measurements. They were also advised to wear loose and comfortable clothing. On the day of the experiment, each participant first underwent a neck ultrasound performed by a radiologist to precisely locate the carotid sinus, which was marked by encircling the area with a non-permanent surgical skin marker for accurate placement of the stimulation chamber. Carotid sinus localization was performed using ultrasound by a single trained radiologist, following a standardized anatomical landmark protocol. This approach minimized inter-operator variability and ensured consistency across participants.

Heart rate (lead II ECG) and chest movements were continuously monitored using a computer-based digital data acquisition system (Power Lab, AD Instruments, Australia) with a sampling rate of 1 kHz, ensuring high-resolution signal recording. Beat-to-beat arterial blood pressure was recorded non-invasively using the NIBP system (CNSystems, Austria), which employs the vascular unloading technique for continuous blood pressure monitoring with a high sampling rate of 200 Hz. After a 10-minute supine rest period, heart rate variability (HRV) was assessed in a supine position within the autonomic function testing (AFT) lab, where room temperature was maintained at 24°C in a noise-free, dark environment. Lab Chart software (AD Instruments) was used for real-time signal acquisition and analysis, including RRI extraction and spectral HRV analysis. Both the PowerLab and CNSystems devices were calibrated according to manufacturer guidelines before each session. All ECG and BP recordings were visually inspected, and segments with motion artifacts or ectopic beats were excluded. Automated filters and manual verification ensured artifact-free intervals were selected for analysis.

For the procedure involving neck chamber stimulation, volunteers were instructed to hold their breath at the end of expiration for 15 seconds to standardize intrathoracic pressure, minimize respiratory influences on autonomic tone, and reduce artifacts in cardiovascular recordings. This step was also included because previous studies often did not control for respiratory modulation, which may have contributed to inconsistencies in their reported outcomes. At the fifth second, a neck chamber suction pressure of −20 mmHg was applied and maintained for 5 seconds, as peak heart rate changes typically occur within 3 to 4 seconds after stimulation. The suction pressure was then released, and participants resumed normal breathing 5 seconds later. Each stimulus was applied with a 60-second interval between applications. The same procedure was repeated for subsequent pressure variations of −40 mmHg, −60 mmHg, −100 mmHg, +20 mmHg, +40 mmHg, +60 mmHg, and +100 mmHg. The choice of negative and positive pressure levels (−20 mmHg to +100 mmHg in 20 mmHg steps) was based on prior human studies and our preliminary work [17]. This range reflects the physiologically relevant pressure required to safely and effectively activate carotid baroreceptors. These increments were selected to provide sufficient resolution for dose-response analysis while minimizing participant burden. The pulse duration of carotid stimulation in the present study was chosen as 5 seconds, as this has been reported as the optimal duration to elicit peak heart rate response in previous studies [2,4,17]. This short stimulation period prevents adaptation of carotid baroreceptors and reduces the risk of counteraction from extracarotid baroreceptors, both of which are essential considerations when estimating BRS. The current study looked at heart rate changes over 10 seconds after stimulation, taking into account that the time it takes for human baroreceptor-cardiac reflexes to respond is between 240 and 475 milliseconds, mainly controlled by the cardiac vagus nerve [2,4,17]. Notably, the most substantial heart rate change typically occurs just before the end of a 5-second pressure pulse [2,4,17]. To ensure that each stimulus produced an isolated and interpretable physiological response, a 60-second interval was maintained between successive stimuli. This time was chosen because earlier research shows that at least 45 seconds is needed for the body's automatic functions and blood flow measures to go back to normal after stimulating the carotid baroreceptors [2,4,17]. The 60-second window thus provided a safe buffer to prevent carryover effects and ensured consistent baseline conditions for each stimulus application.

**Figure 7:**
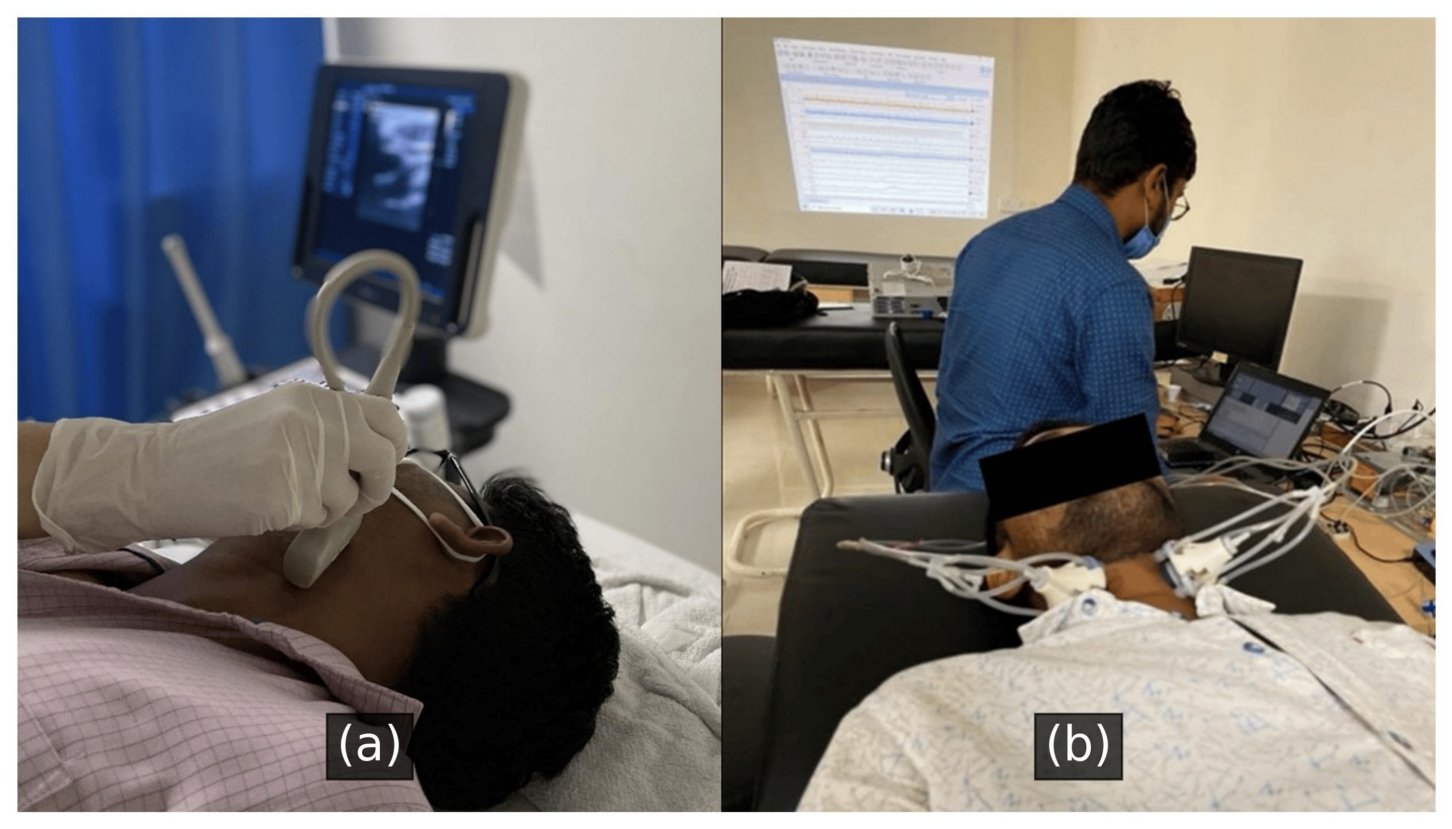
The device arrangement on test volunteers, along with the recording mechanism used in the study (a) Ultrasound-guided localization of the carotid baroreceptor prior to stimulation. A radiologist performs neck ultrasonography to identify the precise location for optimal placement of the stimulation device. (b) Bilateral carotid baroreceptor stimulation using the paired neck chamber device. The volunteer undergoes controlled stimulation while physiological signals, including heart rate and blood pressure, are continuously recorded for analysis.

The order of stimulation sides (left, right, bilateral) and pressure levels (ranging from −100 mmHg to +100 mmHg in 20 mmHg steps) was randomized across participants using a computer-generated sequence to minimize order effects and reduce anticipatory responses. A dedicated team of researchers applied the neck chamber based on pre-marked carotid sinus locations. Participants were continuously monitored for adverse effects such as discomfort, dizziness, or syncopal symptoms throughout the procedure. A structured symptom checklist and verbal confirmation were used after each stimulation. No participant reported any adverse effects during or after the sessions. A separate team performed the ECG and BP signal extraction and analysis, and they were blinded to the stimulation condition to ensure an unbiased interpretation of autonomic and hemodynamic responses. Figure 7 illustrates the device arrangement on test volunteers, along with the recording mechanism.

RRIs, SBP, and DBP were recorded before and during each stimulation.

Statistical Tests

We hypothesized that bilateral carotid baroreceptor stimulation would elicit stronger and more stable autonomic and hemodynamic responses than unilateral stimulation, and that left- and right-sided stimulations would produce distinct effects on heart rate and blood pressure, respectively, due to functional lateralization. To test this, we employed repeated measures ANOVA, post-hoc pairwise testing with Bonferroni correction, nonlinear logistic regression modeling with AIC-based comparisons, and BRS gain analysis. These methods together provide a robust framework to statistically test our lateralization hypothesis.

We conducted a repeated measures analysis of variance (ANOVA) to compare the effects of left, right, and bilateral carotid baroreceptor stimulation on autonomic (RRI) and hemodynamic (SBP and DBP) responses across various graded negative and positive pressure stimuli. This method accounts for within-subject variability and is appropriate for repeated measurements across multiple conditions.

The relationship between CSP and RRI was analyzed using a four-parameter logistic function, applying nonlinear least squares regression to find the best-fitting values. This model was selected because baroreceptor responses usually show an S-shaped curve, and the logistic function helps to determine important values like the point where the response starts, the maximum response, and the rate of change, which are significant in studying baroreflexes. Akaike’s information criterion (AIC) was used to see how well the model fits, and the first derivative of the logistic function was calculated to estimate BRS gain.

The Friedman test was used to compare the highest BRS gain under different stimulation conditions, and then Wilcoxon signed-rank tests with Bonferroni correction were performed to compare pairs. P-values less than 0.05 were seen as statistically significant. P-values <0.05 were considered statistically significant.

Multiple linear regression was used to look at how BRS relates to resting autonomic tone markers (RMSSD, HF power, and LF/HF ratio), checking for significance with p-values and assessing how well the model fits with R². This analysis provided insight into whether baseline autonomic tone acted as a potential confounding factor or predictor of BRS magnitude. Prior studies have often not accounted for this potential confounding factor, which may partly explain the contradictory findings reported in the literature regarding baroreflex asymmetry.

## Results

Comparisons of RRI, SBP, and DBP

**Figure 8:**
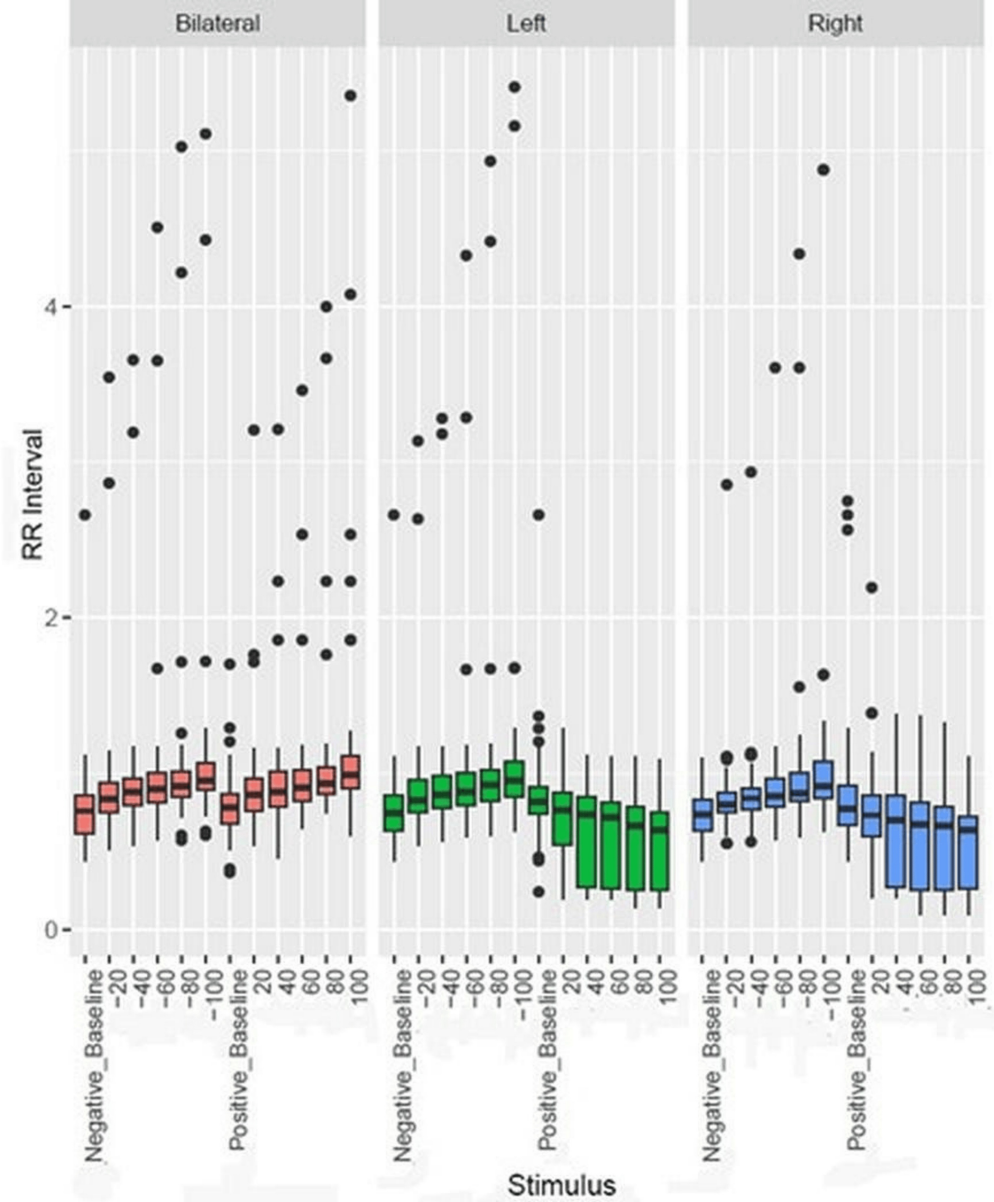
Box plot showing comparison of bilateral, left, and right carotid baroreceptor stimulation on RRI The y-axis denotes the RRI, while the x-axis indicates the stimulation type and intensity. Outliers are shown as individual points. Negative and positive stimuli correspond to neck suction and pressure applications, respectively. RRI, RR interval

**Figure 9:**
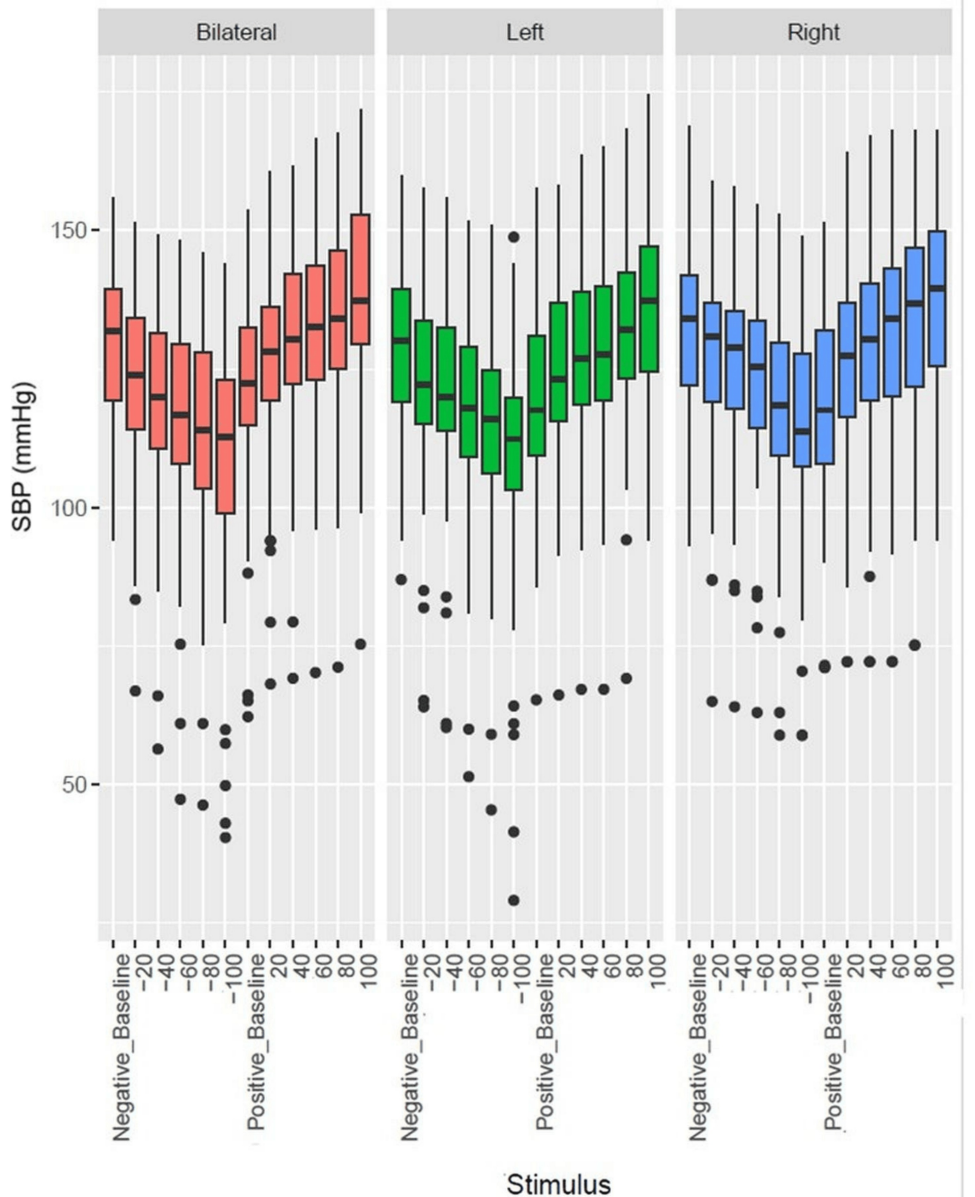
Box plot showing comparison of bilateral, left, and right carotid baroreceptor stimulation on SBP The y-axis denotes the SBP in mm of Hg, while the x-axis indicates the stimulation type and intensity. Outliers are shown as individual points. Negative and positive stimuli correspond to neck suction and pressure applications, respectively. SBP, systolic blood pressure

**Figure 10:**
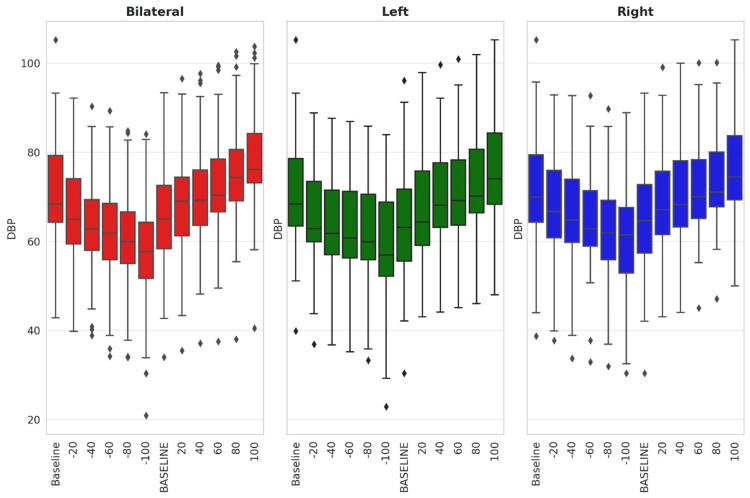
Box plot showing comparison of bilateral, left, and right carotid baroreceptor stimulation on DBP The y-axis denotes the DBP in mm of Hg, while the x-axis indicates the stimulation type and intensity. Outliers are shown as individual points. Negative and positive stimuli correspond to neck suction and pressure applications, respectively. DBP, diastolic blood pressure

Figure 8, Figure 9, and Figure 10 are the boxplots to demonstrate the comparison of the effects of left, right, and bilateral carotid baroreceptor stimulation on RRI, SBP, and DBP, respectively, using graded negative and positive pressure stimuli. At baseline, RRI, SBP, and DBP were similar across conditions. With a graded increase in negative pressure, RRI progressively increased while SBP and DBP progressively decreased. However, positive pressure gradually decreased the RRI while increasing the SBP and DBP.

Figure 8 depicts that bilateral stimulation elicited the most pronounced bradycardia response (increased RRI), followed by left and right stimulation during negative pressure. Conversely, during positive pressure, bilateral stimulation resulted in the smallest reduction in RRI, while left and right stimulation led to more substantial decreases. Statistical analysis using repeated measure analysis of variance across left, right, and bilateral confirmed that bilateral stimulation induced significantly greater autonomic modulation, with left-sided stimulation causing slightly stronger RRI perturbations than right, both of which were statistically significant. (p<0.05)

Similarly, Figure 9 and Figure 10 demonstrated the greatest decrease in SBP and DBP with bilateral stimulation, followed by left and right stimulation during negative pressure. In contrast, positive pressure induced the highest SBP increase with right-sided stimulation, followed by left and bilateral stimulation. For DBP, the most significant change occurred with bilateral stimulation, followed by right and then left carotid stimulation. Overall, these findings suggest that bilateral baroreceptor stimulation produces more stable and uniform changes in SBP and DBP, whereas unilateral stimulation, especially right-sided, induces larger and more variable fluctuations in SBP and DBP, which were statistically significant.

All data points, including those appearing as outliers on boxplots, were included in the statistical analysis to preserve the integrity and variability of the dataset. These points were visually flagged using boxplot conventions (1.5 × IQR), but they did not meet any criteria for exclusion based on protocol deviations or measurement errors. A small subset of participants (n = 4) demonstrated paradoxical responses (e.g., tachycardia in response to negative pressure), which were retained in the final analysis. These cases were not excluded, as they were not related to artifacts or errors but rather reflected individual autonomic variability.

Comparison of difference of RRI, SBP, and DBP from baseline values

Table 1 presents the change in RRI from baseline under graded negative and positive pressures for left, right, and bilateral carotid baroreceptor stimulation. With negative pressure, RRI progressively increased, with the most pronounced bradycardic effect at -100 mmHg across all conditions. No statistically significant difference was observed among the three conditions at -60, -80, and -100 mmHg. However, with positive pressure, RRI progressively decreased, with bilateral stimulation showing a significantly greater tachycardic response compared to unilateral stimulation (p < 0.001). Notably, for left and right stimulation, the decrease in RRI remained smaller and statistically similar across all positive pressure levels (p < 0.001 for bilateral vs. unilateral). These findings indicate that bilateral stimulation results in a significantly stronger tachycardia response under positive pressure, whereas bradycardia effects under negative pressure remain comparable across all stimulation conditions.

**Table 1:** Descriptive statistics for change in RRI from baseline to different negative and positive stimuli according to side *Obtainer using repeated measure analysis of variance across left, right, and bilateral; Bold p-values indicate significant difference. Superscript letters denote significant post-hoc comparisons (Bonferroni-corrected). Values sharing the same letter (e.g., "a") are not significantly different. Values with different letters (e.g., "a" vs. "b") indicate a statistically significant difference (p < 0.05). RRI, RR interval

Side		Negative stimulus					Positive stimulus				
		-20	-40	-60	-80	-100	20	40	60	80	100
Left	Mean	-0.14	-0.18^a^	-0.23^a^	-0.29	-0.37	0.15^a^	0.19^a^	0.21^a^	0.25^a^	0.28^a^
	Median	-0.09	-0.12	-0.13	-0.16	-0.22	0.03	0.06	0.10	0.12	0.15
	SD	0.23	0.30	0.46	0.57	0.67	0.25	0.26	0.28	0.32	0.34
Right	Mean	-0.11	-0.15^a^	-0.19^a^	-0.29	-0.38	0.17^a^	0.24^a^	0.27^a^	0.29^a^	0.32^a^
	Median	-0.07	-0.10	-0.11	-0.15	-0.20	0.05	0.08	0.11	0.13	0.19
	SD	0.25	0.26	0.35	0.58	0.74	0.27	0.37	0.39	0.41	0.46
Bilateral	Mean	-0.16	-0.19^a^	-0.25^a^	-0.29	-0.34	-0.12^b^	-0.17^b^	-0.21^b^	-0.29^b^	-0.41^b^
	Median	-0.09	-0.12	-0.14	-0.16	-0.19	-0.07	-0.10	-0.11	-0.16	-0.21
	SD	0.34	0.36	0.49	0.58	0.59	0.22	0.25	0.30	0.47	0.67
P-value*		0.060	0.024	0.044	0.987	0.659	<0.001	<0.001	<0.001	<0.001	<0.001

Table 2 and Table 3 summarize the changes in SBP and DBP from baseline under graded negative and positive pressure stimuli for left, right, and bilateral carotid baroreceptor stimulation. Under negative pressure, SBP and DBP progressively declined, reaching their lowest levels at -100 mmHg. Bilateral stimulation produced the most pronounced hypotensive effect, followed by left and right stimulation. Statistically significant differences in SBP were observed at -40 mmHg (p = 0.029) and -60 mmHg (p = 0.021), with no significant differences at other negative pressure levels.

**Table 2:** Descriptive statistics for change in SBP from baseline to different negative and positive stimuli according to side *Obtained using repeated measure analysis of variance across left, right, and bilateral; Bold p-values indicate a significant difference. Superscript letters denote significant post-hoc comparisons (Bonferroni-corrected). Values sharing the same letter (e.g., "a") are not significantly different. Values with different letters (e.g., "a" vs. "b") indicate a statistically significant difference (p < 0.05). SBP, systolic blood pressure

Side		Negative stimulus					Positive stimulus				
		-20	-40	-60	-80	-100	20	40	60	80	100
Left	Mean	- 8.02	-10.40^a^	-13.55	-16.62	-22.71	5.79	8.74	10.23	12.84	17.93
	Median	-4.31	-7.19	-9.86	-12.63	-15.79	3.68	7.10	8.51	11.78	17.20
	SD	10.4	11.26	-13.23	14.27	22.62	5.35	6.48	7.18	7.33	9.42
Right	Mean	-5.46	-7.38^a^	-10.35	-15.31	-18.88	7.14	9.60	12.39	15.16	20.49
	Median	-2.62	-4.11	-7.16	-11.30	-13.86	4.99	7.55	9.05	12.10	16.34
	SD	7.56	7.86	8.79	13.61	15.09	7.50	8.50	10.96	12.27	13.87
Bilateral	Mean	-6.73	-10.79^a^	-14.52	-17.34	-24.35	5.49	8.18	10.91	7.80	9.44
	Median	-2.96	-6.05	-8.59	-11.06	-15.95	3.60	6.04	7.83	9.58	13.85
	SD	8.23	13.47	16.44	16.46	22.27	7.74	9.60	10.85	26.48	29.28
P-value*		0.060	0.029	0.021	0.547	0.265	0.327	0.566	0.443	0.122	0.024

**Table 3:** Descriptive statistics for change in DBP from baseline to different negative and positive stimuli according to side *Obtained using repeated measure analysis of variance across left, right, and bilateral; Bold p-values indicate significant difference. Superscript letters denote significant post-hoc comparisons (Bonferroni-corrected). Values sharing the same letter (e.g., "a") are not significantly different. Values with different letters (e.g., "a" vs. "b") indicate a statistically significant difference (p < 0.05). DBP, diastolic blood pressure

Side		Negative stimulus					Positive stimulus				
		-20	-40	-60	-80	-100	20	40	60	80	100
Left	Mean	-5.98^a^	-7.61^a^	-9.03^a^	-10.09	-13.24	3.83^a^	5.90^a^	7.46^a^	9.51^a^	12.86^a^
	Median	-3.48	-5.00	-7.11	-7.98	-10.31	2.00	3.90	5.17	6.99	10.06
	SD	6.99	7.40	7.65	7.64	9.76	4.82	5.81	5.98	6.80	7.85
Right	Mean	-3.75^b^	-5.31^b^	-7.02^a^	-9.58	-11.80	3.99^a^	6.02^a^	7.61^a^	9.52^a^	12.60^a^
	Median	-2.00	-3.83	-5.32	-7.32	-9.24	3.10	4.96	6.08	7.24	9.49
	SD	4.38	4.82	5.03	9.09	9.82	4.12	5.22	5.80	6.26	7.27
Bilateral	Mean	-5.72^a^	-8.04^a^	-9.47^a^	-10.72	-15.78	2.83^b^	5.93^b^	8.00^b^	10.15^b^	12.67^b^
	Median	-3.60	-4.99	-5.93	-7.96	-10.76	2.72	5.02	6.81	8.95	11.66
	SD	6.29	8.20	9.23	9.08	14.95	1.58	3.58	4.64	5.96	6.10
P-value*		0.002	0.002	0.013	0.553	0.073	<0.001	<0.001	<0.001	<0.001	<0.001

For DBP, significant reductions were noted at -20, -40, and -60 mmHg (p < 0.05), with bilateral and left-sided stimulation demonstrating a greater effect than right-sided stimulation. In response to positive pressure, both SBP and DBP increased progressively. SBP exhibited the highest rise with right-sided stimulation, followed by left and bilateral stimulation, whereas DBP showed the greatest increase with left-sided stimulation, followed by right and bilateral stimulation. Statistically significant differences (p < 0.001) were observed across all positive pressure levels, with bilateral stimulation resulting in a slightly attenuated hypertensive response compared to unilateral stimulation. Overall, these findings indicate that bilateral baroreceptor stimulation provides a more stable and controlled BP response, exerting greater hypotensive effects under negative pressure and a milder hypertensive response under positive pressure compared to unilateral stimulation.

The observed bilateral RRI change was 28.4% lower than the predicted additive sum of left and right responses. For SBP, the bilateral reduction was 35.1% less than the combined unilateral values.

Dose-response relationship

**Figure 11:**
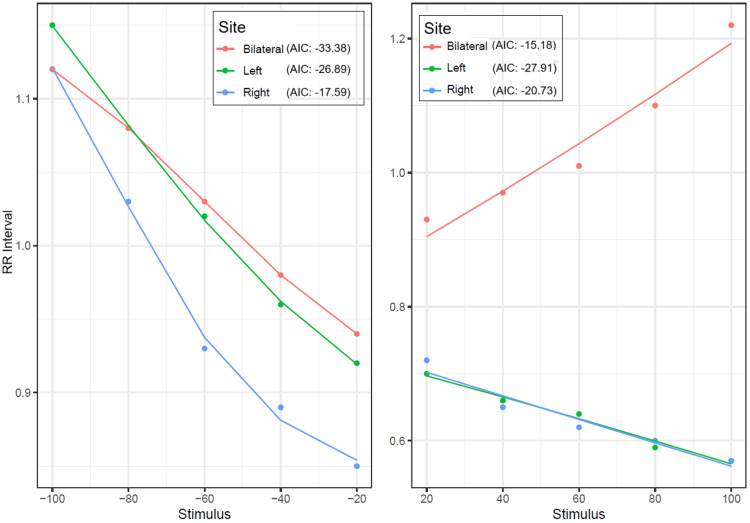
Dose-response relationship of carotid baroreceptor stimulation on RRI across different stimulation sites The left panel represents the response to negative (suction) stimuli, while the right panel depicts the response to positive (pressure) stimuli. The AIC values indicate model fit for each stimulation site, with lower AIC values suggesting a better fit. RRI, RR interval; AIC, Akaike information criterion

**Figure 12:**
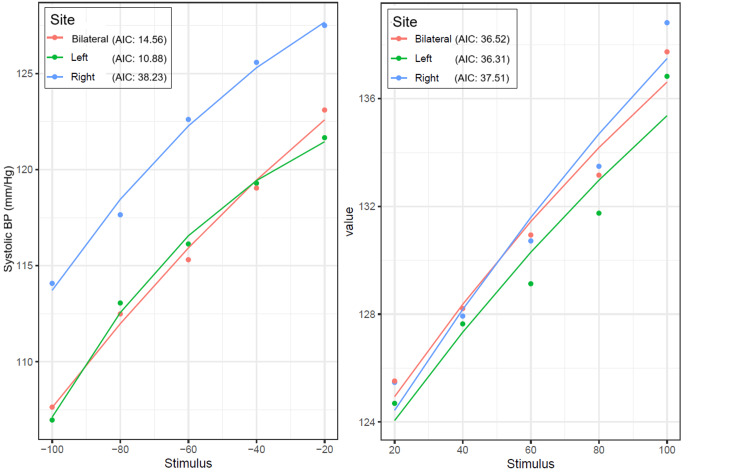
Dose-response relationship of carotid baroreceptor stimulation on SBP across different stimulation sites The left panel represents the response to negative (suction) stimuli, while the right panel depicts the response to positive (pressure) stimuli. The AIC values indicate model fit for each stimulation site, with lower AIC values suggesting a better fit. SBP, systolic blood pressure; AIC, Akaike information criterion

Figure 11 and Figure 12 provide the model fits for RRI and SBP mean response observed for negative and positive stimulii separately. The points are the observed means, while the line plots represent the respective model fits.

A ΔAIC >2 is typically considered indicative of meaningful model improvement, and a difference >4 suggests moderate to strong evidence in favor of the model with the lower AIC. In this context, the ΔAIC of 3.68 for SBP under left vs. bilateral stimulation for negative stimulation indicates a moderately better fit for the left-sided model (AIC: 10.88 and 14.56 for left and right, respectively). On the positive side, both left and bilateral stimulation showed equally good model fits to the data. For the RRI and negative stimulus, the model fit for the bilateral stimulus was the best (AIC: -33.38), while for the positive stimulus, the model fit for the left carotid was the best (AIC: -27.91). The slope coefficients indicating gain were similar for left, right, and bilateral negative stimulations; however, for positive stimulations, the gain was the opposite for bilateral compared to left and right stimulations. The gain coefficients were almost similar across left, right, and bilateral negative as well as positive stimulations.

Baroreceptor sensitivity/gain

**Figure 13:**
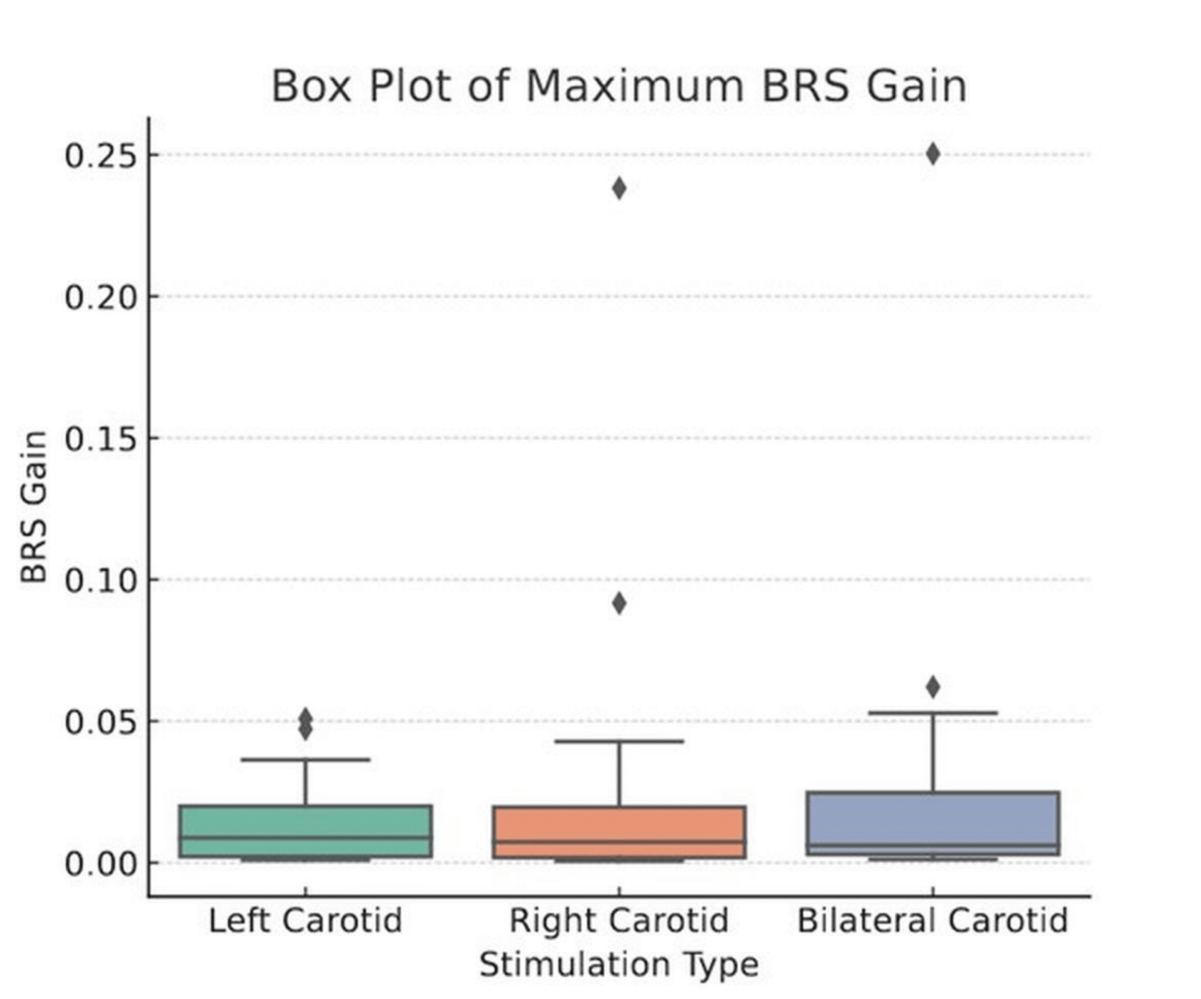
Box plot showing comparison of maximum BRS/gain across left, right, and bilateral carotid stimulation BRS, baroreflex sensitivity

Figure 13 presents the comparison of maximum BRS/gain across left, right, and bilateral carotid stimulations, with mean values of 0.0136 ± 0.0141, 0.0193 ± 0.0409, and 0.0219 ± 0.0423, respectively. BRS gain values are reported in milliseconds per millimeter of mercury (ms/mmHg). A Friedman test (p = 0.4326) indicated no statistically significant differences, and post-hoc Wilcoxon signed-rank tests with Bonferroni correction confirmed no significant pairwise differences. The BRS gain for bilateral stimulation was lower than the sum of the individual left and right responses. 

Multiple linear regression results

Table 4 presents the findings of the multiple linear regression analysis, indicating that baseline autonomic markers, RMSSD, HF power, and the LF/HF ratio, exhibited limited predictive value for BRS, as reflected by low R² values across all models. Left and right BRS showed no significant associations with autonomic markers, while bilateral BRS exhibited a weak trend toward significance for the LF/HF ratio (p = 0.074), suggesting a potential but minor role of sympathovagal balance in bilateral baroreflex regulation.

**Table 4:** Multiple linear regression correlation of BRS measures with HRV parameters p-values < 0.05 indicate significance. HRV, heart rate variability

BRS measure	R²	RMSSD (p-value)	HF (p-value)	LF/HF ratio (p-value)	Significance
Left BRS	0.112	0.32	0.110	0.111	Not significant
Right BRS	0.034	0.925	0.361	0.297	Not significant
Bilateral BRS	0.091	0.858	0.094	0.074	Trending toward significance (LF/HF ratio)

## Discussion

Design and development of the upgraded paired neck chamber

The development of the upgraded paired neck chamber device represents an advancement in non-invasive carotid baroreceptor stimulation technology. The device's design significantly enhances its capability to provide regulated positive and negative pressure stimuli within −100 mmHg and +100 mmHg. This precise modulation is achieved using a pair of diaphragm pumps and solenoid valves, ensuring consistent and reproducible stimulation of the carotid baroreceptors. The use of 3D printing technology for manufacturing the neck chambers, along with a low-shore hardness circumferential ring and silicone-based suction cups, improves ergonomic alignments and ensures a snug fit to the skin. These design factors are essential for reducing discomfort during extended testing sessions.

The device's dual-mode capability, enabling unilateral and bilateral stimulation, provides flexibility in evaluating the functional asymmetry of baroreflex pathways. The device enables targeted stimulation, allowing for more detailed investigations into baroreflex processes and their role in cardiovascular regulation. The incorporation of a Raspberry Pi-based digital control system significantly improves the device's capabilities by enabling automatic pressure modulation and real-time monitoring. This function diminishes operator dependence and enhances the device's utility in both research and clinical environments. The user-friendly computer interface simplifies operation, making it accessible to people with diverse levels of technical proficiency. The system incorporates real-time pressure monitoring, a software-controlled upper-pressure limit (±105 mmHg), and manual emergency stop features to ensure participant safety. The dual-pump mechanism and the soft-sealing chamber interface also reduce mechanical risk during prolonged contact.

In the field of newer therapeutics, such as BAT [18] for resistant hypertension and heart failure, the capacity to execute accurate and consistent baroreceptor stimulation is becoming increasingly vital. Recent clinical research has evidence for the effectiveness of BAT in reducing blood pressure and enhancing heart function [18-20]. The enhanced neck chamber apparatus may function as a significant instrument in the non-invasive evaluation and adjustment of these therapies, thereby facilitating patient selection and treatment refinement. The device's performance in various populations should be validated in future research, and its potential uses in clinical practice as a novel therapy need to be investigated.

Table 5 depicts a comparative summary of existing neck collars and upgraded chamber devices for baroreflex stimulation. The technique for carotid stimulation also differs across studies. Table 5 highlights the evolution from manual, bilateral-only models to advanced, digitally controlled systems enabling unilateral or bilateral stimulation with improved precision and comfort. Most neck collar-based devices deliver bilateral stimulation using sinusoidal waveforms, with a focus on negative pressure only, while positive pressure application remains largely unexplored. In contrast, our study uses a new, locally made neck chamber device that can apply controlled positive and negative pressures to the left, right, or both carotid sinuses separately. This is enabled by two mechanically independent chambers connected to an electro-pneumatic logic control unit, allowing true unilateral stimulation, an advancement over earlier methods. 

**Table 5:** Comparative summary of neck collar/chamber devices for baroreflex stimulation

Device & study	Stimulation type	Target site	Control mechanism
Early Animal Model (Dog) Device [21]	Mechanical pressure (plaster bandage)	Bilateral (dogs)	Manual
Square Section Neck Collar Box [22]	Mechanical pressure via large rigid box	Bilateral only (humans)	Manual
Rapid Quantification Neck Collar [23]	Controlled pressure changes	Bilateral only (humans)	Analog pressure control
Paired Neck Chamber (Simplified Teaching Model) [24]	Mechanical stimulation using paired earphones	Bilateral only (humans)	Manual (via suction and pressure)
Low-Noise Dual Pressure Neck Chamber [25]	Electro-mechanical with noiseless pressure tanks	Both unilateral and bilateral (humans)	Digital embedded system with ARM microcontrollers
Noiseless Variable-Pressure Neck Chamber [16]	Electro-mechanical	Bilateral only (humans)	Microcontroller-based system with touch display and vacuum tank
Strap-Free Suction-Based Neck Chamber [17]	Mechanical square-wave pressure stimulation (±60 mmHg)	Unilateral only (humans)	Manual pump with pressure control and safety valves
Current Upgraded Paired Neck Chamber	Electro-pneumatic positive & negative pressure	Both unilateral and bilateral (humans)	Digital GUI-based (Raspberry Pi, solenoid valves)

The neck chamber device was tested on a sample of over 100 participants with varying neck anatomies, including differences in neck circumference, skin texture, subcutaneous tissue composition, and facial hair. The soft, low Shore hardness interface and dual-chamber suction system made sure it stayed attached and sealed well for everyone, without any major air leaks or loss of pressure. No participant reported discomfort or detachment during the stimulation protocol. This finding highlights the device’s robustness and adaptability for use in diverse populations.

While the current system operates in an open-loop mode, future iterations could incorporate real-time physiological feedback (e.g., heart rate or BP) to enable closed-loop modulation. This would allow the system to dynamically adjust stimulation parameters based on user-specific autonomic responses, thereby improving therapeutic precision and individualization.

Comparison of BRS for laterality of response

The baseline cardiovascular parameters like RRI, SBP, and DBP in this study were similar across left, right, and bilateral conditions, confirming that any observed changes were due to the applied stimuli. Under graded negative pressure, an increase in RRI occurred concurrently with decreases in systolic and DBPs, reflecting enhanced baroreceptor activation that promotes parasympathetic activity and reduces sympathetic output. In contrast, graded positive pressure resulted in a decrease in RRI and increases in both blood pressure measures, indicative of diminished baroreceptor signaling and a subsequent rise in sympathetic activity. These findings align with the physiological role of baroreceptors in maintaining cardiovascular homeostasis. When negative pressure is applied, it stretches the carotid sinus wall, mimicking an increase in blood pressure activating baroreceptors, leading to increased parasympathetic activity, slowing the heart rate. Opposite effects are obtained when positive pressure is applied due to compression of the carotid sinus wall, reducing stretch on baroreceptors, leading to decreased parasympathetic activity and increasing the heart rate [2]. A typical U-shaped response trend in SBP, DBP, and partly RRI box plot visualization underscores the dose-dependent responsiveness of the carotid baroreflex.

The bilateral carotid baroreceptor stimulation elicited the most pronounced bradycardic response under negative pressure and the most controlled tachycardic response under positive pressure compared to unilateral stimulation. Bilateral stimulation activates baroreceptors on both the left and right carotid sinuses simultaneously. This provides a stronger and more synchronized afferent signal to the brainstem compared to unilateral stimulation. The brainstem responds with a more robust increase in parasympathetic activity and a decrease in sympathetic activity, leading to a more pronounced bradycardic response. Bilateral stimulation ensures that both sides of the baroreflex arc are engaged equally, avoiding any asymmetry in neural input that might occur with unilateral stimulation [26]. This balance likely results in a more consistent and stronger bradycardic effect. This finding is consistent with studies by Eckberg et al. [15] and Heusser et al. [27], who reported that bilateral carotid baroreceptor activation produces stronger and more consistent autonomic modulation compared to unilateral stimulation. The ability of bilateral stimulation to control heart rate and blood pressure more effectively than unilateral stimulation has important clinical implications. For example, it could be used in devices or therapies for conditions like hypertension, heart failure, or autonomic dysfunction, where maintaining stable cardiovascular parameters is critical.

Left-sided stimulation exhibits slightly stronger RRI effects than right-sided, possibly due to inherent differences in baroreceptor sensitivity and central signal processing. Variations in anatomical features, such as vessel size, wall elasticity, and receptor density, might make the left carotid more responsive to pressure changes [28]. Additionally, differences in vascular tone and lateralized brainstem integration could further enhance the left-sided response. These local and central factors together explain the observed asymmetry [26]. Previous studies report conflicting results, some support our study finding that the left carotid has a greater influence on cardiac vagal activity [29], while some studies report the right carotid as a better response [9,10] or no significant difference [12]. These discrepancies might have arisen from methodological variations, such as predominantly animal research, small and uneven human samples, inconsistent stimulation protocols, and unaccounted influences of respiration and baseline autonomic tone. Larger, more standardized studies across diverse populations are needed to clarify these findings.

Bilateral stimulation resulted in more consistent and uniform blood pressure modulation, as indicated by smaller standard deviations and reduced variability in DBP and SBP responses compared to unilateral stimulation. This suggests a more stable baroreflex engagement when both carotid sinuses are activated simultaneously. In contrast, right-sided stimulation produced greater fluctuations in both SBP and DBP, possibly reflecting a stronger sympathetic vasomotor component associated with right carotid baroreceptor activation. These differences were statistically significant and are detailed in Table 3, where the magnitude and variability of DBP changes across stimulation conditions are presented. These results are inconsistent with the previous studies. Williamson JW et al. [12] and de Leeuw PW et al. [13] findings are similar to our findings, while several reported left carotid better [9,29] and some reported no difference [10]. This discrepancy may be due to differences in experimental protocols, such as the range of pressures applied or the population studied. However, the ability of bilateral stimulation to mitigate extreme BP changes highlights its potential therapeutic utility in conditions such as hypertension and orthostatic hypotension. Interestingly, a similar pattern is observed at the positive pressure end of the curve, especially under bilateral stimulation, where the response appears to flatten or reverse. This pattern may indicate autonomic saturation, signal damping, or reflex inhibition at higher stimulation intensities, reflecting nonlinear central processing of carotid baroreceptor input.

The analysis of maximum BRS gain across left, right, and bilateral carotid stimulations revealed no statistically significant differences between the stimulation conditions. While the mean values showed a slightly higher gain for bilateral stimulation (0.0219 ± 0.0423) compared to the left (0.0136 ± 0.0141) and right (0.0193 ± 0.0409) stimulations, the Friedman test (p = 0.4326) indicated that these variations were not statistically meaningful. Additionally, post-hoc Wilcoxon signed-rank tests with Bonferroni correction further confirmed the absence of significant pairwise differences.

Our observation is that bilateral stimulation offers more stable autonomic modulation, while left and right stimulation yield distinct effects on cardiac and blood pressure responses. This implies that each patient could receive customized baroreflex activation therapy (BAT). For example, patients who have predominant heart rate issues, such as inappropriate sinus tachycardia, postural orthostatic tachycardia syndrome, or heart failure with preserved ejection fraction, where controlling heart rate is critical, might see better results from stimulation on the left side because it affects the vagus nerve more strongly. In contrast, patients with resistant hypertension may benefit more from right-sided or bilateral stimulation due to more robust blood pressure control. Future research should focus on validating these lateralization-based approaches in clinical settings to support the development of personalized BAT protocols.

No signs of habituation or adaptation were observed during repeated stimulation trials within a session. The use of brief pressure applications and adequate recovery intervals (60 seconds) between stimuli likely prevented desensitization of baroreceptor responses.

Baroreceptor sensitivity/gain

In our study, the comparison of BRS gain across left, right, and bilateral carotid stimulations did not reveal any statistically significant differences (Friedman test, p = 0.4326), a result further supported by post-hoc Wilcoxon signed-rank tests. These findings are consistent with prior human studies. Furlan et al. reported no significant differences in RRI, blood pressure, or muscle sympathetic nerve activity (MSNA) across right, left, and bilateral carotid stimulations [10]. Similarly, Williamson and Raven observed comparable BRS values across stimulation sites, although bilateral stimulation appeared to exhibit a non-additive, inhibitory response relative to the sum of unilateral effects. In contrast, animal studies, such as Kawada et al. [11], have demonstrated greater baroreceptor sensitivity on the right side, highlighting potential interspecies differences in baroreflex integration. Conversely, some human studies have reported significant lateralization effects favoring either side [13,29], which may be attributed to variability in baseline physiological factors across studies. Notably, resting autonomic tone is a key determinant influencing BRS and may account for the inconsistent findings in the literature.

While the mean BRS values for bilateral stimulation appeared numerically higher than unilateral stimulation, these differences were not statistically significant. We have therefore refrained from drawing physiological conclusions based on these trends. Instead, we recognize this issue as an area warranting further investigation, particularly in larger or more stratified cohorts that may better capture subtle interindividual variability or central integrative mechanisms.

Dose-response relationship

A four-parameter logistic model was used to fit the CSP-RRI relationship due to its ability to accurately represent the nonlinear sigmoidal behavior of baroreflex responses. The model parameters allowed estimation of the maximum gain (BRS) using the first derivative at the curve's steepest point. Model fit was evaluated using AIC, with a ΔAIC >10 considered a significant improvement over simpler models.

For the RRI, using both sides of stimulation with negative pressure worked best (AIC: −33.38), indicating it engages the baroreflex more effectively. In contrast, with positive pressure, the left side stimulation had the best fit (AIC: −27.91), suggesting that different areas affect heart responses in specific ways. Under positive pressure, stimulating the left side worked best, indicating that different areas can affect heart responses in specific ways. Interestingly, the gain (slope) values were alike for all stimulation sites during negative pressure, but bilateral stimulation had a different gain direction under positive pressure, possibly indicating complicated central processing or a response from the sympathetic nervous system.

For SBP, the best fit under negative pressure was observed with left-sided stimulation (AIC: 10.88), followed closely by bilateral stimulation. Under positive pressure, model fits for left and bilateral stimulation were comparably strong. Gain estimates were relatively consistent across all sites and conditions, indicating that blood pressure regulation may be less site-dependent than heart rate modulation.

These findings reinforce the notion that baroreflex responses vary by stimulation site and pressure polarity and that bilateral and unilateral stimulation may engage distinct autonomic pathways. The differences in how well the models work and their responsiveness show that it is important to think about which side is being stimulated and the direction of the pressure when designing experiments and using baroreflex stimulation for treatment.

Multiple linear regression results

We selected RMSSD, HF power, and the LF/HF ratio as markers of resting autonomic tone due to their physiological relevance. RMSSD and HF reflect parasympathetic activity, while LF/HF provides a sympathovagal balance index. SDNN, while useful, is more sensitive to total variability and influenced by factors beyond autonomic tone. Similarly, LF power alone lacks specificity, particularly in short-term recordings [4]. Variance Inflation Factor (VIF) values for all predictors were below 2, confirming low multicollinearity in the regression model.

The multiple linear regression analysis revealed that RMSSD, HF power, and LF/HF ratio, markers of resting autonomic tone, had minimal influence on BRS, with low R² values across all models. Given that these factors were expected to act as potential confounders in the comparison of BRS across left, right, and bilateral stimulations, their lack of significant association suggests that resting autonomic tone does not substantially bias the observed differences. The near-significant association (p = 0.074) between the LF/HF ratio and bilateral BRS may suggest that baseline sympathovagal balance influences the sensitivity of integrated baroreflex responses, particularly during simultaneous bilateral input. While the effect was modest in this healthy cohort, it could have greater clinical relevance in patients with dysregulated autonomic tone, such as those with hypertension or heart failure, where baseline sympathetic predominance might blunt or exaggerate baroreflex gain. Moreover, R² values were low across regression models; this is consistent with the multifactorial regulation of BRS. VIF analysis indicated no significant multicollinearity among predictors (all VIFs <2.0). The trend-level association between the LF/HF ratio and bilateral BRS (p = 0.074) was not corrected for multiple comparisons and should be interpreted as exploratory. Given the modest sample size and known physiological variability, these non-significant results may reflect limited statistical power rather than the absence of biological relevance.

Table 6 shows the comparative summary of key human and animal studies evaluating lateralized carotid baroreflex stimulation, highlighting the study population, stimulation methods, and primary findings, including the present study using an indigenously developed paired neck chamber device.

**Table 6:** Comparative summary of studies evaluating carotid baroreflex laterality

Study	Population	Stimulation type	Key findings
Tafil-Klawe et al. [9]	30 healthy adults (24-38 yrs)	Neck capsules (−60 to +60 mmHg)	Right-sided activation → stronger cardiac reflex; Left → more blood flow acceleration; functional asymmetry proposed.
Williamson & Raven [12]	20 healthy men (22-34 yrs)	Unilateral neck collar (−65 to +50 Torr)	No major left-right difference in HR; Bilateral response < sum of individual sides; Suggests inhibitory interaction in humans.
Williamson et al. [11]	10 healthy adults (22-36 yrs)	Sustained neck pressure (~25 mmHg)	Left-sided stimulation → greater muscle SNA; suggests left-sided dominance for sympathetic regulation.
Furlan et al. [10]	12 healthy volunteers (mean 32 yrs)	Sinusoidal suction (0.1 Hz, up to −50 mmHg)	Right-sided stimulation → stronger cardiac modulation; No MSNA asymmetry; the bilateral response was similar to the right stimulation
de Leeuw et al. [13]	295 hypertensive patients	Implanted BAT device	Right-sided stimulation → better BP reduction than left/bilateral; supports right-preference in clinical BAT.
Salman et al. [29]	9 hypertensive rats	Electrical ADN stimulation (1-40 Hz)	Left & bilateral stimulation → stronger BP & HR reduction vs. right; left-dominant central integration in rats.
Present Study (2025) (AIIMS Nagpur & IIT Jodhpur)	108 healthy young adults (mean age ~23 yrs)	Digitally controlled paired neck chambers (−100 to +100 mmHg)	Bilateral stimulation → strongest, most consistent HR (RRI↑) and BP (SBP/DBP↓) modulation under negative pressure; Positive pressure → greater BP ↑ with right side; Slightly stronger bradycardia with left; No statistically significant BRS difference; bilateral effects were non-additive, suggesting central integration.

Our finding that bilateral stimulation produced a response smaller than the sum of left and right responses is consistent with Williamson & Raven [12], who observed inhibitory central integration during bilateral carotid baroreceptor activation. This suggests that bilateral afferent input may engage inhibitory interneurons or result in neural saturation within the NTS or related centers. In contrast, de Leeuw et al. [13] found that right-sided stimulation more effectively lowered blood pressure, particularly in hypertensive patients. This difference might be due to the groups studied, such as healthy people in our study compared to hypertensive patients in de Leeuw et al., which could greatly affect how the baroreflex responds and its side effects because of changes in the body's normal functions, blood vessel structure, or reduced feedback in patients with health issues. Furthermore, methodological differences, such as the use of sinusoidal suction vs. sustained pressure, influence baroreceptor adaptation and neural encoding. A previous study by Furlan et al. [10] used sinusoidal suction (like 0.1 Hz, rhythmic) to imitate natural blood pressure changes, while the sustained suction or pressure used in our device triggers the strongest reflex responses. These approaches differ in how baroreceptor adaptation, afferent firing patterns, and central habituation are engaged. Using sustained negative pressure in our study might lead to stronger and more reliable reflex responses because it gives a longer signal and reduces the effects of becoming less sensitive over time. There are species-specific differences in human and animal studies, such as differences in carotid sinus anatomy, baroreceptor density, afferent nerve composition, and baseline autonomic tone between species that may explain conflicting findings. For instance, rabbits have more segmental and diffuse baroreceptor fields [30], while humans have more localized carotid sinus structures, potentially affecting stimulus transduction. In addition, autonomic setpoints and baroreflex gain vary considerably across species due to evolutionary cardiovascular adaptations [31].

Our results, showing the strongest RRI responses with left-sided stimulation and the strongest BP reductions with right-sided stimulation, are in agreement with the hypothesis of functional laterality in baroreflex modulation.

Strengths and limitations

This study provides a comprehensive comparison of left, right, and bilateral carotid baroreceptor stimulation utilizing a novel paired neck chamber device; it enables precise, non-invasive, and controlled stimulation, ensuring safer and more accessible assessments. With a relatively larger sample size and rigorous methodological controls, including corrections for baseline autonomic activity and respiratory effects, the findings are robust and reliable. These results have direct clinical implications for BAT and autonomic modulation in conditions like hypertension, heart failure, and autonomic dysfunction, paving the way for personalized therapeutic approaches. Given individual variations in baroreceptor sensitivity, personalized autonomic modulation therapies incorporating genetic, hemodynamic, and neurophysiological profiling may enhance treatment outcomes.

The present study has several limitations. These findings are based on acute baroreflex responses in healthy young adults and may not directly generalize to older adults or clinical populations with autonomic dysfunction, cardiovascular disease, or structural vascular changes. The non-invasive neck chamber technique used in this study does not allow for precise measurement of the actual pressure transmitted to the carotid sinus or the extent of baroreceptor stretch, which may have influenced the observed responses. Direct neural recordings of autonomic activity (such as MSNA or vagal nerve activity) were not performed, limiting insights into the central neural processing of baroreflex responses. The study focused on acute baroreflex responses, and long-term adaptations to repeated carotid stimulation were not explored. The non-additive effect of bilateral stimulation suggests complex central integration, but the exact neural mechanisms need to be further explored. Future research should examine chronic adaptations, particularly activity-dependent plasticity within the nucleus tractus solitarius, which is the key integrative center for baroreceptor inputs. Adaptive changes in autonomic set-point regulation and central gain control over time should also be investigated to optimize individualized neuromodulation strategies. Neuroimaging and electrophysiological techniques may help elucidate how prolonged or lateralized stimulation modulates central autonomic processing. This study did not conduct a gender-based subgroup analysis. Since there are known differences between genders in autonomic tone and BRS, future studies should look at these responses separately to better understand possible gender-specific mechanisms and treatment options. Formal usability testing and structured participant feedback were not part of the present study. However, no adverse experiences or complaints were reported. Future work should include systematic usability evaluations to assess comfort, ease of use, and participant acceptance, especially in the context of longer-term applications or closed-loop therapeutic use. Although the physiological response patterns observed align with prior literature, the present study did not include benchmark validation against gold-standard methods such as pharmacological baroreflex testing or established mechanical collar systems. This represents an important limitation. We plan future studies to directly compare the device's output with existing standard devices. 

The ability to non-invasively evaluate individual autonomic and hemodynamic responses to lateralized baroreceptor stimulation could support personalized patient selection and therapy optimization in BAT. For instance, patients with predominant heart rate dysregulation (e.g., HFpEF or POTS) may benefit from left-sided stimulation, while those with hypertension may respond better to right-sided or bilateral stimulation. This device may serve as a screening or titration tool to guide and refine BAT implementation in clinical practice. 

## Conclusions

The novel paired neck chamber device enabled precise, non-invasive, and controlled carotid baroreceptor stimulation, contributing to advancements in baroreflex assessment methodologies. This study demonstrated that bilateral stimulation induced the strongest bradycardic response under negative pressure and a more controlled tachycardic response under positive pressure, indicating enhanced autonomic modulation compared to unilateral stimulation. Among unilateral stimulations, left carotid stimulation had a slightly stronger effect on cardiac function, while right carotid stimulation exerted a greater influence on blood pressure regulation. BRS did not significantly differ across stimulation sites. The weak trend of baseline HRV influencing bilateral BRS suggests that baseline autonomic tone, particularly the balance between sympathetic and parasympathetic activity, likely has little to no impact on how BRS works in these experiments. These findings, observed in healthy young adults using a novel non-invasive paired neck chamber device, enhance our understanding of the physiological underpinnings of baroreflex function. While the study suggests lateralized baroreceptor influences and supports the feasibility of precise side-specific stimulation, we acknowledge that these results cannot be directly extrapolated to clinical populations. This study lays the groundwork for a non-invasive, safe, and easy-to-use design for individualized baroreflex assessment in both research and medical settings, offering a potential platform to guide personalized therapies for hypertension, heart failure, and autonomic disorders such as dysautonomia. Future studies involving patients with cardiovascular or autonomic dysfunctions are essential to validate these observations and explore their potential relevance in guiding mechanical baroreflex-based interventions, such as mechanical BAT. Future studies should focus on long-term adaptations, neuroplasticity, and central baroreflex integration to refine therapeutic strategies for cardiovascular regulation.
